# Synthesis and Degradation Behavior of Poly(glycerol sebacate)-Isophorone Diisocyanate Scaffolds Reinforced with Hydroxyapatite for Biomedical Applications

**DOI:** 10.3390/polym18020304

**Published:** 2026-01-22

**Authors:** Aleksandra Korbut, Agnieszka Sobczak-Kupiec, Monika Biernat, Sonia Zielińska

**Affiliations:** 1 Department of Polymer Engineering and Technology, Faculty of Chemistry, Wroclaw University of Science and Technology (WUST), Wyb. Wyspianskiego 27, 50-370 Wroclaw, Poland; 2 Department of Materials Engineering, Cracow University of Technology, Jana Pawła II Av. 37, 31-864 Cracow, Poland; agnieszka.sobczak-kupiec@pk.edu.pl; 3 Biomaterials Research Group, Ceramic and Concrete Division in Warsaw, Łukasiewicz Research Network Institute of Ceramics and Building Materials, Postępu 9, 02-676 Warsaw, Poland; monika.biernat@icimb.lukasiewicz.gov.pl

**Keywords:** poly(glycerol sebacate), hydroxyapatite, scaffolds, biomaterials, chemical crosslinking, incubation

## Abstract

Poly(glycerol sebacate) (PGS) is a biodegradable elastomer with high potential for tissue engineering. However, its limited structural stability and degradation control restrict broader biomedical applications. This study presents an integrated fabrication strategy for highly porous PGS-IPDI scaffolds reinforced with two types of hydroxyapatite of distinct origin (HAP_B and HAP_ICMB). By combining low-temperature urethane crosslinking with thermally induced phase separation and salt leaching, we obtained scaffolds with interconnected micro–macroporous architectures and exceptionally high porosity (up to 98%). The comparative incorporation of phase-pure nanometric HAP_B and biphasic HAP_ICMB enabled the identification of composition-dependent differences in water uptake, structural stability, and mineralization tendencies. Furthermore, degradation behavior was systematically evaluated in four physiologically relevant media (PBS, SBF, artificial saliva, Ringer’s solution), revealing distinct degradation pathways associated with each environment. The results provide new insight into how hydroxyapatite type and incubation medium collectively govern the long-term performance of chemically crosslinked PGS-based scaffolds.

## 1. Introduction

The most important element in tissue engineering is tissue culture, a process that involves the propagation of cells under controlled conditions, allowing precise control over their growth environment. Tissue culture is carried out in vitro on specially prepared three-dimensional structures, called tissue scaffolds, which play a key role in cell development [1]. The main purpose of these structures is to retain the cells on their surface, facilitate their development, and support newly formed cells. Furthermore, scaffolds are designed to maintain specific physical and mechanical properties both during the growth of new cells and after implantation into the recipient’s body [2,3].

Materials used in tissue engineering should be as close as possible to natural tissues in terms of their physical and mechanical properties [4,5]. Polymers such as poly(glycolic acid), poly(lactic acid) [6,7,8,9], polycaprolactone [10,11,12], or collagen [13,14,15] are most commonly used to create scaffolds. These polymers promote cell adhesion to the scaffold surface and cell proliferation because they structurally resemble tissue [4].

Poly(glycerol sebacate) (PGS) is a biocompatible and biodegradable elastomeric polyester synthesized from glycerol and sebacic acid. This polymer has attracted significant attention in biomedical applications due to its remarkable mechanical properties, controlled degradation rates, and excellent biocompatibility [16,17]. These characteristics make PGS an ideal candidate for tissue engineering, drug delivery systems, and regenerative medicine [18,19].

The most common method of poly(glycerol sebacate) (PGS) crosslinking is thermal curing [20]. During this process, unreacted carboxyl and hydroxyl groups react with each other to form a three-dimensional polymer network. However, this type of crosslinking requires demanding conditions, including high temperatures and long processing times; therefore, alternative methods have been investigated to facilitate the process. One such approach involves crosslinking the polymer with diisocyanates [21,22,23]. This method chemically crosslinks poly(glycerol sebacate) through a reaction between the free hydroxyl groups of the polymer and the isocyanate groups. As a result, the process time is significantly reduced, and the need for high temperatures is eliminated. Furthermore, the introduction of urethane groups into the polymer structure improves tensile strength compared to pure poly(glycerol sebacate). This enhancement may be attributed to the formation of covalent crosslinks and an increased contribution of interchain hydrogen bonding. Moreover, diisocyanate crosslinking does not alter the physical form of PGS at room temperature or at human body temperature; the polymer retains its elastomeric properties due to its amorphous nature. With respect to susceptibility to enzymatic degradation, polymers containing urethane groups exhibit greater resistance over time than those containing only ester groups, as the urethane moieties hinder access to the ester bonds, thereby slowing the degradation process [24,25].

Poly(glycerol sebacate) has a mechanical strength that doesn’t quite match the parameters of living tissues. To enhance the mechanical properties and stability of PGS, crosslinking agents such as isophorone diisocyanate (IPDI) [26,27] are employed. IPDI is a well-known aliphatic diisocyanate that provides rigidity and resistance to hydrolysis, resulting in a more robust polymer network. The introduction of IPDI into the PGS matrix creates a three-dimensional crosslinked structure, which significantly improves the tensile strength, elongation, and resistance to enzymatic degradation of the polymer.

To improve the bioactivity of polymeric materials, composites incorporating ceramic phases such as hydroxyapatite can be developed; this approach has shown promising results in bone tissue engineering [28]. Calcium hydroxyapatite, Ca_10_(PO_4_)_6_(OH)_2_, enables the formation of stable bonds with bone tissue, exhibits osteoconductive properties, and does not induce acute inflammatory reactions in living organisms [29]. A common feature of bone and hydroxyapatite is calcium phosphate, which in its crystalline form closely resembles the mineral phase of bone tissue. Moreover, synthetic calcium hydroxyapatite typically does not contain ionic impurities such as fluoride, magnesium, or sodium, which may be present in calcium phosphate occurring in the human body. The method used to synthesize hydroxyapatite significantly influences the structure, specific surface area, and degree of crystallinity of the resulting material [30].

Composite scaffolds based on biodegradable polymers combined with hydroxyapatite (HAP) have been widely investigated as bone-mimicking materials due to their ability to couple controlled degradation with enhanced bioactivity and mechanical performance [31,32]. Incorporation of HAP into these polymers has been shown to significantly improve compressive modulus, stiffness, and dimensional stability of the scaffolds, while simultaneously promoting osteoconductivity and cell-mediated mineralization [33]. For example, PCL/HAP [34] and PLA/HAP composites [35] exhibit reduced degradation rates compared to neat polymers due to restricted polymer chain mobility and decreased water diffusion, allowing prolonged mechanical support during bone regeneration. In PLGA/HAP systems [36], HAP additionally buffers acidic degradation by-products, mitigating local pH drops and improving cytocompatibility. In elastomeric PGS/HAP composites [37], chemical crosslinking of the polymer network combined with inorganic reinforcement enables fine control over mechanical properties and degradation kinetics, resulting in gradual scaffold resorption synchronized with extracellular matrix deposition and new bone formation.

Moreover, progressive exposure or partial dissolution of HAP during polymer degradation leads to sustained release of calcium and phosphate ions, which further stimulate osteogenic differentiation and accelerate tissue maturation. Collectively, these examples demonstrate that polymer–HAP composite scaffolds enable precise tuning of biodegradation behavior while maintaining mechanical integrity and biological functionality essential for effective bone tissue regeneration. In contrast, non-biodegradable synthetic polymers such as poly(2-hydroxyethyl methacrylate) (PHEMA), poly(N-(2-hydroxypropyl)methacrylamide) (PHPMA) [38], and poly(methyl methacrylate) (PMMA), as well as conductive polymers, have been used in orthopaedical applications due to their biocompatibility, reproducible mechanical properties, and ease of processing; however, their lack of biodegradability necessitates surgical removal and limits their use in tissue regeneration. These limitations have motivated the development of biodegradable polymer–HAP composite scaffolds that combine temporary mechanical support with bioactivity and controlled biodegradation, enabling progressive replacement of the scaffold by newly formed bone tissue.

The aim of this study was to design, synthesize, and comprehensively characterize highly porous poly(glycerol sebacate) based scaffolds crosslinked via urethane chemistry and modified with hydroxyapatite, intended for potential biomedical and tissue engineering applications. The work focused on developing a controlled two-step approach involving the synthesis of a pPGS prepolymer, its chemical modification with isophorone diisocyanate (IPDI), and subsequent formation of a crosslinked elastomeric network with a well-defined porous architecture. The interaction of the developed materials with physiologically relevant degradation media was systematically investigated to assess their stability, degradation mechanisms, and surface evolution under simulated biological conditions. To comprehensively evaluate the stability, degradation mechanisms, and bioactivity of the developed PGS-IPDI scaffolds and their hydroxyapatite-reinforced composites, four physiologically relevant incubation media—PBS, SBF, artificial saliva, and Ringer’s solution—were selected. Each medium represents a distinct physiological environment and enables the identification of different degradation pathways: neutral hydrolysis (PBS), mineralization and bioactivity (SBF), chemically accelerated degradation (artificial saliva), and calcium-driven interactions characteristic of extracellular fluid (Ringer’s solution). The combined use of these solutions allows for a multidimensional assessment of long-term scaffold performance, providing a robust basis for determining their suitability for bone tissue engineering applications. By combining spectroscopic, microscopic, thermal, and physicochemical analyses, this study sought to provide an integrated understanding of how polymer–filler interactions and environmental conditions govern the long-term performance of PGS-IPDI-based scaffolds.

Based on the distinct physicochemical characteristics of the two hydroxyapatite powders, we formulated the following hypotheses:

(1) HAP_ICMB, a biphasic HAP/β-TCP material subjected to high-temperature calcination and exhibiting increased structural porosity and defect density, would induce greater water uptake within the PGS-IPDI scaffolds, intensify ion-exchange interactions with the incubation media, thereby accelerating medium-dependent degradation processes, and provide a higher density of nucleation-active sites, promoting enhanced calcium-phosphate deposition.
(2) HAP_B, a phase-pure, nanometric hydroxyapatite obtained via wet precipitation under boiling conditions and characterized by a denser and more uniform microstructure, would integrate more homogeneously into the polymer matrix, result in lower water sorption and reduced degradation rates, and support a more gradual and controlled mineralization behavior.

These hypotheses form the basis for the comparative evaluation of scaffold hydration, structural evolution, bioactivity, and degradation profiles.

## 2. Materials and Methods

### 2.1. Materials

Sebacic acid, glycerol, and isophorone diisocyanate were purchased from Aldrich. Sodium chloride with 400–500 µm grain size from P.P.H “STANLAB” Sp.J. (Poland, Lublin) was used. Tetrahydrofuran and 1,4-dioxane were purchased from Eurochem BGD (Poland, Tarnów). As filler, two different types of hydroxyapatite particles were used: pure, nanometric hydroxyapatite (HAP_B) obtained by a wet precipitation method at boiling point [39,40,41] and hydroxyapatite synthesized by a wet precipitation method at 50 °C (HAP_ICMB) [42]. Obtained pure, nanometric HAP_ICMB hydroxyapatite was calcinated at 1200 °C. The resulting micrometric HAP_ICMB has the following composition: 93.77 wt% HAP, 5.69 wt% β-TCP, 0.54 wt% CaO.

### 2.2. Synthesis and Characterization of PGS-Based Materials

^1^HNMR spectra were recorded with an NMR AvanceTM400 MHz spectrometer (Bruker, Billerica, MA, USA) using CDCl_3_ as solvent and tetramethylsilane as an internal standard.

The Attenuated Total Reflectance-Fourier Transform Infrared Reflectance spectra (ATR-FTIR) were recorded with a Nicolet iZ10 spectrometer (Thermo Scientific, Waltham, MA, USA) equipped with a Smart iTR™ diamond ATR accessory. The spectra were acquired with a resolution of 4 cm^−1^ in the range of 4000–500 cm^−1^ (32 co-added scans).

Density of the scaffolds was measured using the buoyancy method by a Hildebrand Electronic Densimeter H-300S (Wendlingen am Neckar, Germany). To estimate the porosity, the following Equation (1) was used:

(1) $$\Phi_{p}=\left[1-\frac{\rho_{sc}}{\rho_{b}}\right]\cdot 100\%$$

where Φ*_p_* is the porosity, *ρ_sc_* is the scaffold density, and *ρ_b_* is the bulk density of the composite scaffold. At least ten repetitions for each sample, the average value and standard deviation were taken as a result.

The thermogravimetric analysis was performed with a TGA/DSC1 Mettler Toledo thermobalance (Columbus, OH, USA). Samples were heated at a rate of 10 °C/min from 25 to 900 °C under a 60 mL/min nitrogen flow.

The DSC measurements were performed using the Mettler Toledo DSC1 system (Columbus, OH, USA), coupled with a Huber TC 100 intracooler (Peter Huber Kältemaschinenbau SE, Offenburg, Germany) (mass: ~5.5 mg; nitrogen flow N2: 60 mL/min; heating or cooling rate: 10 °C/min, temperature range: −70 °C to 250 °C. After the heating cycle, the samples were thermally equilibrated at 250 °C for 5 min and cooled down to −70 °C. A second heating scan was also performed.

Wettability of the samples was measured using a PG-X contact angle goniometer (Testing Machines, Inc., New Castle, DE, USA), with at least ten repetitions for each sample; the average value and standard deviation were taken as a result. As the wetting liquid, distilled water was used.

Water uptake measurements involved measuring the mass of foams over time, starting with weighing a dry sample before placing it in demineralized water to determine its initial mass (*m*_0_). Measurements were taken after 15, 30, 45, 60, 120 min, and 24 h. The experiment was repeated three times for each foam from each series.

The water absorption of porous materials was defined as the ratio of the mass of the wet sample to the mass of the dry sample and was determined according to Equation (2):

(2) $$W.U.=\frac{m_{w}-m_{0}}{m_{0}}\cdot 100\%$$

where *W.U.* is the water uptake (%), *m*_0_ is the mass of the dry sample (g), and *m_w_* is the mass of the water-soaked sample (g).

Statistical analysis for water uptake and porosity was performed using Student’s *t*-test to compare two independent groups. Differences were considered statistically significant at *p* < 0.05. All results are presented as mean ± standard deviation. One-way ANOVA with Tukey’s post-hoc test was used to analyze the effect of the incubation medium on changes in the measured parameters during incubation (pH, weight loss, and wettability), with statistical significance defined at *p* < 0.05.

Elemental analysis was performed on a Thermo Scientific Flash Smart instrument (Waltham, MA, USA), determining the content of the following elements: CHN/O. BBOT (Thermo Fisher Scientific, Waltham, MA USA) was used as a standard. Samples were analyzed before degradation, after degradation, and after extraction. Extraction was performed in acetone, the solvent was evaporated, and the resulting extract was analyzed.

The microstructure of the obtained porous composites was tested by scanning electron microscopy with field emission (Nova NanoSEM 200, FEI, Eindhoven, The Netherlands). Before the study, the samples were covered with a conductive material (25 nm gold film) using a sputter coater (EM SCD500, Leica, Vienna, Austria). Imaging of composites was visualized in high vacuum conditions using an ETD detector (Everhart–Thornley detector combined with Nova NanoSEM 200) at 15 kV accelerating voltage and at magnifications of 1000×.

Prepolymer pPGS was synthesized via reduced-pressure polycondensation. Synthesis and characterization details of pPGS were described in our previous paper [40]. Briefly, sebacic acid and glycerol were heated to 130 °C and stirred mechanically for 48 h. The structural characterization of the obtained prepolymer was defined based on analysis of ^1^H NMR and FTIR spectra.

**Figure 1:**
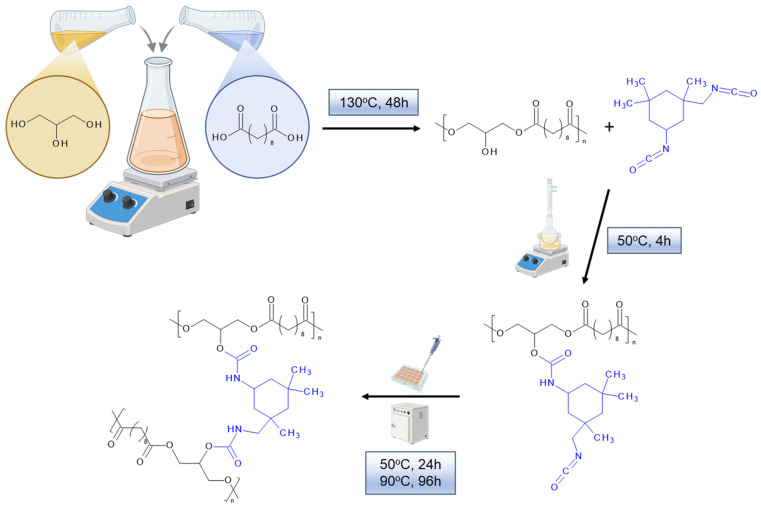
Chemical reaction of poly(glycerol sebacate) prepolymer modifications and subsequent crosslinking with isophorone diisocyanate.

Chemical modification of poly(glycerol sebacate) prepolymer with isophorone diisocyanate was shown in Figure 1 and described below.

In a round-bottom flask, 18.00 g of pPGS was placed and dissolved in 90 mL of anhydrous tetrahydrofuran. Then, 7.60 mL (8.06 g, 36.2 mmol) of isophorone diisocyanate was added to the solution in small portions. The contents of the flask were heated at 50 °C under a reflux condenser for 4 h with continuous stirring on a magnetic stirrer. After this time, the solvent was evaporated on a rotary evaporator to give an oily liquid in 75% yield.

### 2.3. PGS-Based Scaffold Preparation

PGS-based porous scaffolds were obtained by thermally induced phase separation combined with porogen leaching (TIPS-SL [43]). At first, PGS-IPDI was dissolved in 1,4-dioxane to prepare a 20 wt% solution. For composites, hydroxyapatite (HAP_B or HAP_ICMB) was added (15 wt% relative to the polymer weight), and the resulting suspension was stirred vigorously and ultrasonicated until a homogeneous system was obtained. Next, into each well of a silicon 37-well plate, 1.50 g of NaCl with a grain size of 400–500 µm as porogen and previously prepared solution (PGS-IPDI) (or dispersion PGS-IPDI/15HAP_B or PGS-IPDI/15HAP_ICMB) were added, and then was left in the plate in the air for 24 h. Samples were frozen and freeze-dried for 24 h. After this, the samples were crosslinked: at 50 °C for 24 h and then at 90 °C for 96 h. Finally, the foams were washed with a large quantity of deionized water while controlling the conductivity of the washing medium and dried at 50 °C to constant weight. The sample composition is described in Table 1.

### 2.4. Investigation of the Interactions Between the Incubation Fluids and the Samples

To simulate the effects of the synthesized biomaterials in living organisms, we decided to use solutions of artificial biological fluids, such as simulated body fluid (SBF), artificial saliva, Ringer’s solution (representing the composition of extracellular fluid), and PBS (physiologically buffered saline) (Table 2) [44]. Incubation studies were then conducted, and changes in the materials were carefully monitored as they degraded in an environment simulating the body’s environment. Careful monitoring and analysis of the foams allowed us to determine whether the material degradation process resulted in the precipitation or release of components that could negatively impact cellular equilibrium. The foams were incubated for 56 days in the four physiological solutions mentioned above. The solutions were prepared by dissolving the appropriate components in distilled water. The incubation process was carried out at 37 °C. Regular analyses of pH and mass change were performed at predetermined intervals (0, 7, 14, 21, 28, 35, 42, 49, and 56 days) to investigate the interactions between the incubation fluids and the samples.

## 3. Results and Discussion

### 3.1. Prepolymer Synthesis, Chemical Modification and Crosslinking

**Figure 2:**
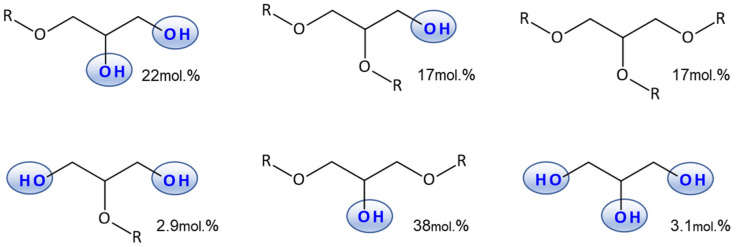
Composition of prepolymer pPGS glyceridic units derived from ^1^H NMR spectrum detailed analysis.

The structural parameters of the prepolymer, divided into glyceride units, were determined based on the ^1^H NMR spectrum, according to the methodology proposed by Perin [45] and Liu [46] and are presented in Figure 2.

The number average degree of polymerization (Dpn) calculated from the ^1^H NMR spectrum is 7.4, which corresponds to an Mn value of 1900 g/mol (calculation was performed based on Hölter et al. [47] model for linear and branched polymers), The molecular weight of the prepolymer is close to the values reported in the literature for an analogous synthesis method [48]. The COOH groups conversion degree is 0.82, the degree of conversion of α-hydroxyl groups is 0.5, and the conversion of β-hydroxyl groups is 0.11. The actual ratio of glyceride residues to sebacic acid residues in the prepolymer is 5.45, determined from the ^1^H NMR spectrum.

**Figure 3:**
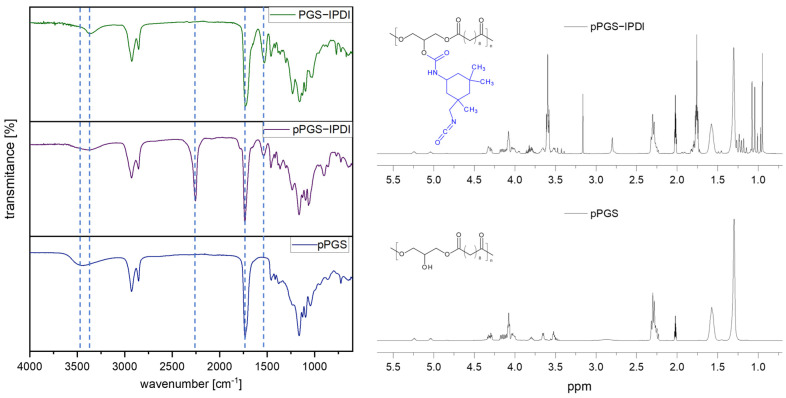
FTIR (left) and ^1^H NMR (right) spectra of pPGS prepolymer and its modification product, pPGS-IPDI. Dot lines on the FTIR spectra refer to the characteristic absorption bands studied in the paragraph below.

The molar ratio of IPDI to pPGS in the prepolymer was calculated from the ^1^H NMR spectrum on the basis of the integration ratio of the characteristic bands for both components. For pPGS, this was the band located at 2.3–2.4 ppm, corresponding to the methylene groups in the sebacic acid residues adjacent to the carbonyl groups. For IPDI, the band located at 0.9–1.15 ppm, corresponding to three methyl groups, was selected. The actual molar ratio of IPDI to pPGS determined in this way is 0.48:1. The ^1^H NMR spectra of pPGS prepolymer and modified pPGS-IPDI are shown in Figure 3.

The ATR-FTIR spectra of pPGS, pPGS-IPDI, and the crosslinked PGS-IPDI confirm the expected chemical evolution from a free hydroxyl groups containing polyester prepolymer to an isocyanate-functional intermediate and, finally, to a urethane-crosslinked network. The spectrum of pPGS is dominated by features typical for an aliphatic polyester: a broad O–H stretching band in the 3200–3600 cm^−1^ region assigned to terminal hydroxyl groups, C–H stretching bands of aliphatic chains around 2950–2850 cm^−1^ and a strong ester carbonyl band at ~1740 cm^−1^. After chemical modification with IPDI, the new urethane-related absorption bands appear. In particular, bands attributable to N–H stretching (~3350 cm^−1^) and the amide II region (~1540 cm^−1^, arising from N–H bending coupled with C–N stretching) become evident, indicating formation of urethane linkages. In addition, the appearance of an isocyanate band near ~2270 cm^−1^ in pPGS-IPDI is consistent with the presence of free NCO groups in the intermediate, as expected for an IPDI-modified prepolymer intended for subsequent crosslinking. In the crosslinked PGS-IPDI product, the isocyanate band at ~2270 cm^−1^ disappears, confirming further reaction of –NCO groups during network formation. Simultaneously, urethane-related bands remain present.

TIPS enables the fabrication of three-dimensional, bulk scaffolds with open, interconnected porosity and without limitations on thickness. The pores are typically larger (tens to hundreds of micrometers) and more isotropic, which supports cell migration into the scaffold, vascularization, and the diffusion of nutrients and metabolites. Electrospinning most often results in dense fibrous mats in which the pores are small and poorly interconnected, and cell colonization occurs mainly at the surface. The mats are usually thin (<1 mm) and multilayered.

A key advantage of foams produced via the TIPS technique is their better suitability for load-bearing applications (e.g., bone or cartilage tissue). Electrospun mats exhibit poor resistance to delamination. Three-dimensional scaffolds provide a more uniform stress distribution and better compressive resistance. The TIPS technique allows homogeneous dispersion of the inorganic phase and control of the porosity through the selection of solvent and porogen. In electrospun mats, the inorganic phase within the fibers may be distributed non-uniformly, and the filler content is often limited (due to rheological issues). In tissue engineering applications, TIPS-derived scaffolds are better suited for the regeneration of bone and cartilage, or soft tissues requiring bulk scaffolds. Electrospinning is more advantageous in applications where nanofibrous topography and surface-level ECM mimicry are critical (e.g., skin, membranes) [49].

### 3.2. Physical Properties of PGS-Based Scaffolds

**Figure 4:**
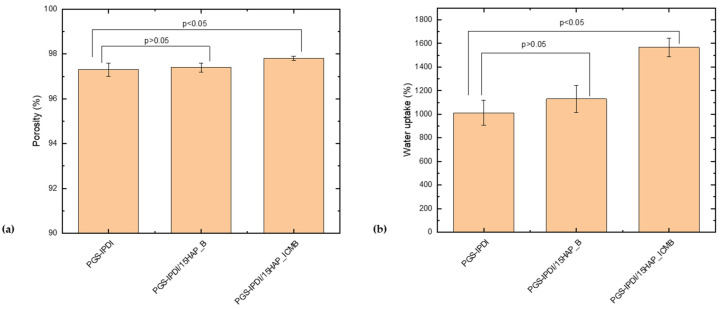
(**a**) Porosity and (**b**) water uptake of PGS-IPDI-based foams. Data are expressed as mean ± SD. Statistical significance was determined using Student’s *t*-test; *p* < 0.05 was considered significant.

All tested PGS-IPDI foams are characterized by very high and comparable porosity, ranging from approximately 97% to 98% (Figure 4a), regardless of the presence or type of hydroxyapatite used. Slightly higher porosity values are observed for the composite foams, particularly PGS-IPDI/15HAP_ICMB. Statistical analysis indicates that the difference between the reference and the PGS-IPDI/15HAP_B sample is not significant (*p* > 0.05), whereas the difference between the reference and the PGS-IPDI/15HAP_ICMB sample is significant (*p* < 0.05). Overall, the porosity remains consistently high across all formulations.

Although the total porosity remains at a similar, very high level for all foams, the type of hydroxyapatite used clearly affects water absorption capacity. The increased water absorption in composite samples can be attributed to the increased hydrophilicity of the material resulting from the presence of the ceramic phase and the more developed microporosity of the pore walls, which promotes more intense water binding and retention. Statistical analysis indicates that the difference between the reference and the PGS-IPDI/15HAP_B sample is not significant (*p* > 0.05), whereas the difference between the reference and the PGS-IPDI/15HAP_ICMB sample is significant (*p* < 0.05). This suggests that the HAP_ICMB filler substantially increases water absorption, while HAP_B has a smaller and statistically insignificant effect. Pure, non-calcinated hydroxyapatite (HAP_B) exhibits lower water uptake because its microstructure is relatively dense and uniform, offering limited pathways for capillary absorption. In contrast, calcination at 1200 °C performed for HAP_ICMB partially decomposes HAP into β-tricalcium phosphate (β-TCP) and calcium oxide (CaO), which introduces structural defects, intergranular porosity, and microcracks. The β-TCP phase further enhances water penetration by creating additional microvoids during initial dissolution [50]. Mixed HAP/β-TCP powders exhibit higher surface energy, contributing to an increased affinity for water. As a result, the calcined HAP_ICMB complex material displays significantly higher water uptake compared with phase-pure HAP_B.

The particularly high water absorption of PGS-IPDI/15HAP_ICMB foams may be beneficial for biomedical applications, such as tissue scaffolds, where a high capacity to absorb body fluids and support nutrient transport is desired. The higher the water absorption value, the more satisfactory the effect can be achieved.

**Figure 5:**
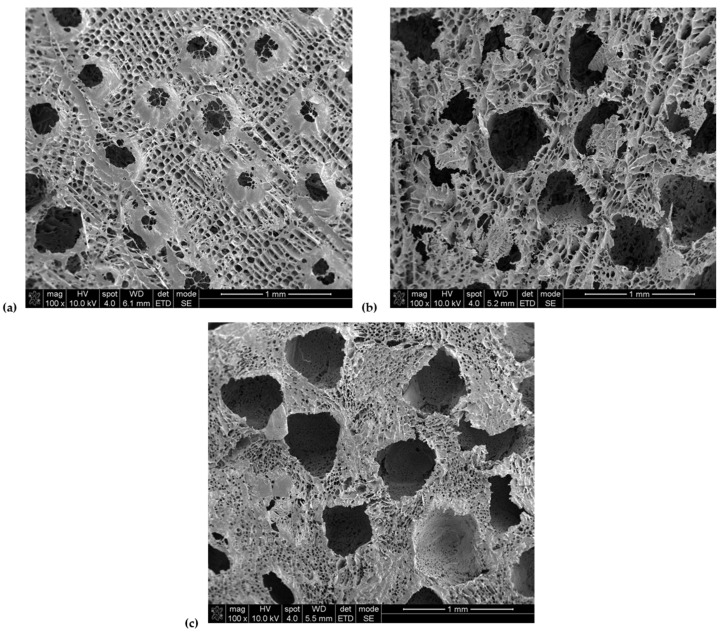
Microscopic photographs of the obtained foams: (**a**) PGS-IPDI, (**b**) PGS-IPDI/15HAP_B, and (**c**) PGS-IPDI/15HAP_ICMB.

Based on the SEM images presented in Figure 5, it can be concluded that all tested PGS-IPDI foams are characterized by a well-developed, open porous structure, typical for materials obtained by the porogen leaching method. In the case of the PGS-IPDI reference sample (Figure 5a), irregular, interconnected pores with relatively large diameters are observed, the size of which is mainly in the range of several hundred micrometers. This size is consistent with the fraction of salt used (400–500 µm), which confirms the effectiveness of the porogen mapping process and its elimination. Additionally, the presence of numerous micropores within the pore walls is observed, increasing the overall porosity of the material. This was a result of the sublimation of 1,4-dioxane. For the PGS-IPDI/15HAP_B foams (Figure 5b), macroscale porosity corresponding to the size of the salt used is retained, but the structure is more irregular. The pores exhibit greater shape variation, and their edges are less smooth, which may be attributed to the presence of hydroxyapatite particles in the polymer matrix.

Despite local deformations, the dominant macropore size still falls within the 400–500 µm range, and the structure remains open and interconnected, which is important for fluid transport and potential cellular colonization. The most uniform and regular macropore structure is observed in the PGS-IPDI/15HAP_ICMB foams (Figure 5c). The pores are clearly separated, with well-defined shapes and diameters similar to salt crystals. At the same time, extensive microporosity of the pore walls is visible, suggesting favorable dispersion of the ICMB hydroxyapatite and a more stable structure formation process. As a result, this material combines high macropore homogeneity with developed secondary porosity. The macropore size in all tested foams corresponds to the size of the salt used (400–500 µm). Differences between samples primarily result from the presence and type of hydroxyapatite, which influences the regularity of the structure and morphology of the pore walls without altering the basic porosity scale imposed by the porogen.

The water contact angle measurements provide insight into the evolution of surface wettability of PGS-IPDI and HAP-containing composites during long-term incubation in physiologically relevant degradation media (Table 3). All materials exhibited initial contact angle values exceeding 110°, confirming the inherently hydrophobic nature of the PGS-IPDI matrix [51]. The incorporation of 15 wt% hydroxyapatite did not significantly reduce the initial hydrophobicity, suggesting that the HAP particles were either well embedded within the polymer matrix or insufficiently exposed at the surface prior to degradation. After 56 days of incubation, a clear dependence of surface wettability on the incubation medium was observed. In PBS, SBF, and Ringer’s solutions, most samples showed an increase in the water contact angle, indicating enhanced surface hydrophobicity. This phenomenon may be attributed to surface restructuring processes, such as the preferential degradation or leaching of more polar segments, leading to the exposure of hydrophobic domains.

Additionally, the adsorption of ions or the formation of surface deposits, particularly in SBF and Ringer’s solution, may contribute to altered surface chemistry and reduced surface energy. In contrast, incubation in artificial saliva consistently resulted in a marked decrease in contact angle for all samples, with the effect being more pronounced for HAP-containing composites. Artificial saliva is a complex medium containing organic components and ions that can promote surface hydration, adsorption of polar species, or partial exposure of hydrophilic HAP particles as the polymer matrix undergoes degradation. The presence of hydroxyapatite likely enhances this effect by providing hydrophilic and ionically active sites, thereby increasing surface wettability after prolonged exposure. Differences between the two HAP-containing systems suggest that the method of hydroxyapatite synthesis influences surface evolution during degradation. The PGS-IPDI/15HAP_ICMB composite exhibited a higher initial contact angle and a more pronounced hydrophobic response in SBF, while still undergoing substantial hydrophilization in artificial saliva. This behavior may reflect differences in particle dispersion, interfacial interactions, or HAP accessibility at the surface [52].

Statistical analysis confirmed that the degradation environment significantly affects the water contact angle in all material systems; however, the strength of this effect decreases with the incorporation of hydroxyapatite. The reference (PGS-IPDI) material without HAP exhibited the strongest dependence on the incubation medium (*p* ≪ 0.001, R^2^ ≈ 0.7), indicating high sensitivity to environmental conditions. The addition of HAP_B reduced the effect size while maintaining statistical significance (*p* < 0.001, R^2^ ≈ 0.60), whereas samples containing HAP_ICMB showed a further attenuation of the environmental influence, resulting in a moderate but still significant effect (*p* = 0.007, R^2^ ≈ 0.49). No statistically significant differences were observed between PBS, SBF, and Ringer’s solution for any material system (*p* > 0.05), indicating that these media exert comparable effects on surface wettability. In contrast, A. Saliva consistently induced the lowest contact angle values and was the only medium responsible for statistically significant differences. The magnitude of the A. Saliva-induced effects decreased in the order: no HAP > HAP_B > HAP_ICMB, demonstrating that hydroxyapatite addition reduces the probability of pronounced surface wettability changes, with HAP_ICMB providing the strongest stabilizing effect.

### 3.3. Thermal Properties of PGS-Based Scaffolds

Thermogravimetric analysis (Table 4) demonstrates that incubation for 56 days in physiological degradation media (PBS, SBF, artificial saliva, and Ringer’s solution) affects the early stages of thermal degradation of PGS-IPDI–based foams, while the high-temperature stability remains largely preserved for most of the analyzed systems. For unfilled PGS-IPDI, incubation leads to a decrease in the onset degradation temperatures (T_−wt%_ and T_−10wt%_), particularly pronounced for samples incubated in artificial saliva. This indicates partial hydrolytic scission of ester/urethane linkages, resulting in lower molecular weight fractions that volatilize earlier. Despite this shift, T_−50wt%_ values and the second inflection point remain comparable to the undegraded material, suggesting that the main polymer backbone retains its thermal robustness. Residual mass at 900 °C remains high, consistent with char formation typical of chemically crosslinked glycerol-based polyesters [20,53,54]. In composite foams containing 15 wt% HAP, the residual mass at 900 °C is largely attributed to the inorganic hydroxyapatite phase, which is thermally stable and does not undergo decomposition under the applied TGA conditions. Therefore, the higher residue observed for HAP-filled samples cannot be directly associated with enhanced thermal stability of the polymer matrix. For both HAP_B- and HAP_ICMB-filled foams, incubation in degradation media results in a reduction of the onset decomposition temperatures relative to the non-degraded samples. This effect is most pronounced for samples incubated in artificial saliva, indicating a more intensive degradation of the polymer matrix in this environment. Nevertheless, the main degradation step, reflected by T_−50wt%_ values and the second DTG inflection point, remains largely unchanged regardless of the degradation medium or HAP type. This behavior suggests that degradation predominantly affects the polymer chains in the early stages of decomposition, likely associated with hydrolysis at the material surface, while the thermal stability of the main polymer network is preserved. The presence of HAP may additionally act as a physical barrier, limiting degradation propagation into the bulk of the material.

Glass transition temperatures (Table 5) determined during the second heating cycle were used for comparative analysis, as this step eliminates the influence of thermal history and physically bound moisture, providing a more reliable measure of structural changes induced by degradation. For neat PGS-IPDI, T_g_ increases after incubation in PBS, SBF, artificial saliva and Ringer’s solution compared to the reference sample, indicating reduced chain mobility, most likely due to preferential leaching of low-molecular-weight species and an increase in the effective crosslink density of the remaining polymer network. In contrast, PGS-IPDI/15HAP_B foams exhibit, for all incubation media, except artificial saliva, a decrease in T_g_ after incubation, suggesting enhanced segmental mobility associated with degradation-induced weakening of polymer–filler interactions and partial release of HAP particles, which may increase free volume within the matrix. An opposite behavior, similar to that described for the neat samples, is observed for PGS-IPDI/15HAP_ICMB composites, where T_g_ increases after incubation, implying that stronger interfacial interactions provided by the HAP_ICMB and selective loss of mobile polymer fractions lead to a more constrained polymer network. Artificial saliva induces a distinct response in all systems, resulting in a pronounced increase in T_g_ regardless of filler presence, which is in agreement with other results observed for that incubation medium.

### 3.4. Investigation of the Interactions Between the Incubation Fluids and the Samples

To determine the content of individual elements in the obtained materials, elemental analysis was performed, determining the following elements: carbon, hydrogen, nitrogen, and oxygen. Reference samples were analyzed before incubation, after incubation, and after extraction. Extraction was performed in acetone for reference samples without hydroxyapatite. The foams were weighed and placed in 0.5 mL of acetone for 24 h, covered. After this time, the foams were removed from the solution, allowed to dry, and the vials containing the extract were opened to evaporate the solvent. The foams were weighed again, and the resulting extract was planned to be used for elemental analysis, but due to the small amount, this was not possible, making it impossible to determine the elemental composition of the substances present in the polymer matrix in an uncrosslinked form. The small mass loss of the foams after extraction (1.39–3.11%) indicates a high degree of crosslinking of the produced materials.

Comparing theoretical (Table 6) and experimental values (Table 7), it can be assumed (reaction—Figure 1) that approximately 0.5 mole of diisocyanate per mole of PGS is present. The experimental composition of the PGS-IPDI sample before degradation is similar to the theoretical one, particularly with respect to carbon content. At the same time, slightly lower H%, O%, and N% values were observed, which may indicate incomplete conversion of functional groups, the occurrence of side reactions, or heterogeneity in the crosslinked foam structure.

After incubation in solutions simulating a physiological environment (PBS, SBF, Artificial Saliva, Ringer’s solution), a slight increase in carbon and nitrogen content was observed, with relatively stable hydrogen and oxygen contents. These changes suggest selective degradation of the oxygen-rich polyester segments of PGS, leading to a relative enrichment of the material in more stable urethane segments derived from IPDI. These results confirm that the degradation process occurs preferentially in the ester part of the material, while the urethane groups exhibit greater resistance to hydrolytic conditions.

Significantly greater differences in elemental composition were observed after the extraction process, which led to a significant decrease in the content of all analyzed elements, particularly carbon and oxygen. This indicates the removal of low molecular weight, non-crosslinked, or weakly bonded material fragments, primarily derived from PGS. The obtained data confirm the complex nature of the PGS-IPDI foam structure and the significant impact of both degradation conditions and extraction processes on its chemical composition.

**Figure 6:**
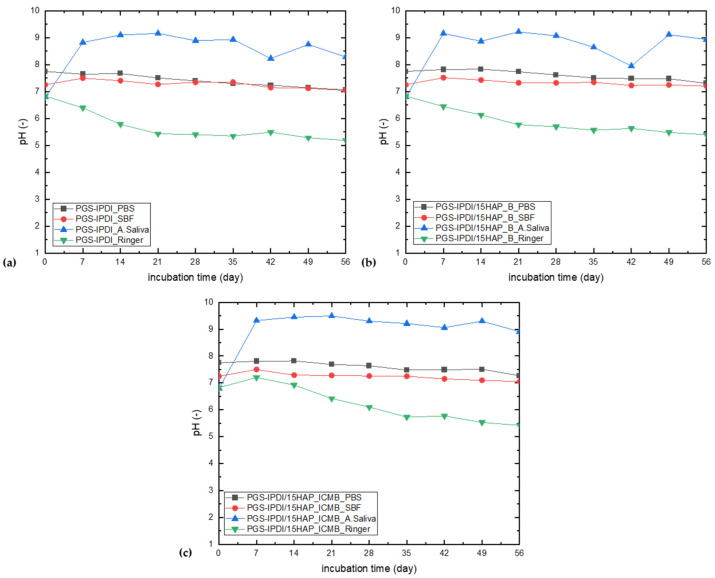
Changes in solution pH depending on foam incubation time: (**a**) PGS-IPDI foams, (**b**) PGS-IPDI/15HAP_B foams, and (**c**) PGS-IPDI/15HAP_ICMB.

The analysis of pH changes in solutions during the incubation of PGS-IPDI and PGS-IPDI_HAPB foams in the incubation fluids (Figure 6) indicates an influence of both the incubation environment and the modification of the material composition on the course of the degradation process. For both kinds of materials, only a slight decrease in pH over time was observed in PBS and SBF solutions, maintaining values close to neutral. This indicates a relatively mild degradation and limited release of acidic hydrolysis products, which is beneficial from the perspective of potential biomedical applications. In the case of artificial saliva, the pH remained distinctly alkaline throughout the degradation period, showing only minor fluctuations over time. This behavior can be primarily attributed to the release of amine-containing degradation products, which may increase the alkalinity of the medium. The presence of these basic compounds dominates the pH evolution, outweighing any buffering effects of the artificial saliva itself. The most dynamic pH changes were observed in Ringer’s solution, where all types of foams experienced a systematic decrease in pH during incubation. This effect is particularly evident in the initial stage of degradation and indicates the intensive release of hydrolysis products from the PGS polyester segments, leading to acidification of the environment. Comparison of the two materials shows that the PGS-IPDI_HAPB foam is characterized by a somewhat milder pH change, especially in PBS and SBF, suggesting a stabilizing effect of the hydroxyapatite additive on the degradation process. The presence of this component may limit the hydrolysis rate and neutralize some of the acidic degradation products, contributing to greater chemical stability of the material under conditions simulating a physiological environment.

Across all incubation media (PBS, Ringer solution, SBF, and artificial saliva), the post-incubation pH response exhibited pronounced medium dependence. One-way ANOVA revealed significant differences in pH among material groups in PBS, Ringer solution, and artificial saliva, where the reference samples differed significantly from both HAP_B and HAP_ICMB containing materials, while no statistically significant differences were detected between the two hydroxyapatite variants. In contrast, incubation in SBF did not result in significant pH differences between groups, although a trend toward group separation was observed. Overall, these results indicate that hydroxyapatite incorporation markedly influences pH modulation in most incubation environments, while HAP_B and HAP_ICMB demonstrate comparable buffering behavior across all tested conditions.

**Figure 7:**
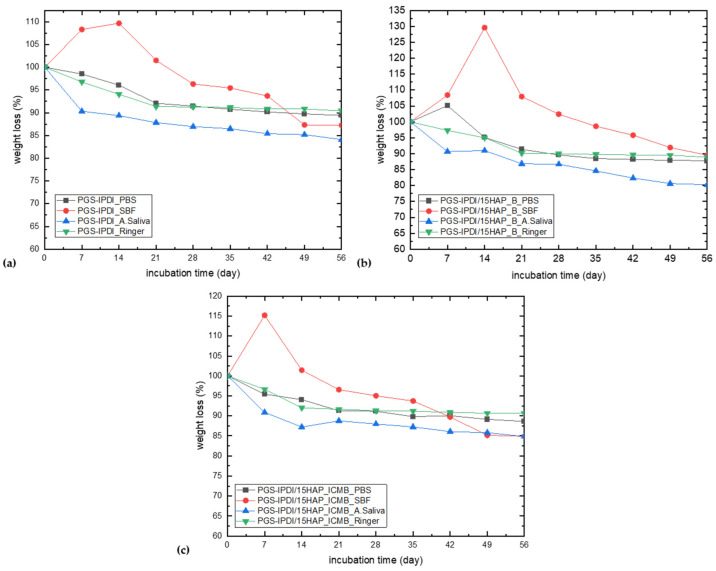
Changes in the mass foams depending on incubation time: (**a**) PGS-IPDI foams, (**b**) PGS-IPDI/15HAP_B foams, and (**c**) PGS-IPDI/15HAP_ICMB.

Changes in the foam’s mass during incubation are presented in Figure 7. The reference PGS-IPDI system exhibits an overall, gradual decrease in mass over time in all incubation media, indicating progressive degradation and leaching of low-molecular-weight components. The most pronounced mass changes are observed in SBF, where an initial mass increase occurs, followed by a clear mass loss. This initial increase suggests uptake of ions or precipitation of inorganic species from SBF onto or into the polymer matrix [55]. In the SBF environment, nucleation and growth of mineral deposits—mainly calcium phosphate phases of an apatite-like character—may occur both on the surface and within the pores of the material. The precipitation of these inorganic phases leads to a real increase in sample mass, enhancing the observed swelling effect. After reaching a maximum mass, a gradual decrease follows, resulting from dominant hydrolytic degradation and leaching of polymer degradation products into the surrounding medium. In solutions that do not provide conditions favorable for mineralization, mass changes are less pronounced and proceed in a more monotonic manner. Comparison of the different foam types indicates that neat PGS-IPDI foams are characterized by a relatively stable mass change profile and a slower degradation rate, which reflects a more compact polymer network structure. In contrast, foams with the addition of HAP_B exhibit the highest initial mass increase in SBF, indicating the highest bioactivity of that mineral filler. PGS-IPDI/15HAP_ICMB foams show an intermediate behavior—the observed mass increase is less abrupt, and the subsequent degradation proceeds in a more uniform manner.

Across all tested incubation media—PBS, Ringer solution, SBF, and artificial saliva—the statistical analyses demonstrated that the incorporation of HAP_B or HAP_ICMB generally did not induce significant changes in mass loss relative to the reference samples. In PBS, SBF, and artificial saliva, the one-way ANOVA results showed no statistically significant differences among the groups, and Tukey post-hoc comparisons confirmed the absence of meaningful pairwise effects, indicating comparable degradation behavior across all formulations. The additive influenced mass loss only in the Ringer’s solution: samples containing HAP_B exhibited significantly higher mass loss than both the reference and HAP_ICMB materials, while the latter did not differ from the control. Overall, the collective findings indicate that material stability is largely unaffected by the additives in most media, with HAP_B exerting a measurable degradative effect solely under Ringer incubation conditions.

The obtained results clearly demonstrate that both the chemical composition of the foams and the nature of the degradation environment, especially the presence of calcium and phosphate ions in SBF, significantly affect the kinetics of swelling, mineralization, and degradation of the material.

In interpreting the in vitro degradation results obtained in this study, it is important to acknowledge their implications and limitations with respect to potential in vivo performance. The four media selected—PBS, SBF, Ringer’s solution, and artificial saliva—represent distinct aspects of the physiological environment and therefore enable the identification of different degradation pathways relevant to clinical scenarios. However, none of these simplified systems fully reproduces the complexity of in vivo degradation, which is influenced not only by hydrolytic cleavage of ester bonds but also by enzymatic activity, continuous fluid exchange, immune response, local pH gradients, and mechanical loading [56]. In bone tissue regeneration, scaffolds must maintain structural integrity during the early phase following implantation while gradually degrading in a manner coordinated with new tissue formation; failure to balance these processes can compromise regenerative outcomes [57,58]. The degradation profiles observed here, characterized by selective hydrolysis of polyester segments and medium-dependent mineral deposition, suggest that the PGS—IPDI network retains sufficient stability over the 56-day period to support initial tissue ingrowth. The presence of hydroxyapatite fillers may further promote osteoconductivity through enhanced ion exchange and nucleation of calcium phosphate phases, which is consistent with recent findings reporting improved bone regeneration performance of HAP-reinforced composites. Nevertheless, confirming appropriate alignment between scaffold degradation and the rate of new bone deposition requires biological validation beyond in vitro incubation studies. Therefore, future work will involve in vitro studies with osteoblast-like cells to assess cellular response, matrix remodeling, and mineralization behavior, followed by in vivo evaluation in a bone defect model to fully determine translational relevance and to optimize degradation kinetics under physiological loading conditions [59].

## 4. Conclusions

We investigated the interactions between the incubation fluids and the samples of PGS-based scaffolds (PGS-IPDI and their composites containing 15 wt% of two types of hydroxyapatite). The TIPS-SL method and crosslinking strategy enabled the fabrication of materials with very high porosity (97–98%), an open and interconnected pore structure, and a high degree of crosslinking, which ensured their chemical and thermal stability. The incorporation of hydroxyapatite does not disturb the pore architecture but modifies the sorption behavior and degradation process.

It was shown that degradation predominantly occurs within the polyester segments of PGS, whereas the urethane linkages are generally more responsible for maintaining the structural and thermal integrity of the materials. The type of incubation medium plays a crucial role in determining changes in surface properties, pH evolution, and mass change kinetics, with artificial saliva being the most aggressive environment. The presence of hydroxyapatite, regardless of its method of synthesis, enhances water uptake and promotes mineralization in simulated body fluid, confirming the bioactive potential of the composites.

The obtained results confirm that the investigated materials are promising candidates for bone tissue engineering applications, offering the possibility of further tailoring their properties through compositional and structural modifications.

## Figures and Tables

**Table 1:** List and description of the investigated samples.

Sample Name	PGS-IPDI Content (wt%)	Apatite Content (wt%)
PGS-IPDI	100	-
PGS-IPDI/15HAP_B	85	15
PGS-IPDI/15HAP_ICMB	85	15

**Table 2:** Buffer compositions used to study the incubation of synthesized polyester urethane foams.

Buffer	Composition	Quantity [g/L]
PBS	NaCl	8.00
	Na_2_HPO_4_	1.15
	KH_2_PO_4_	0.20
	KCl	0.20
SBF	NaCl	8.04
	MgCl_2_ · 6H_2_	0.31
	NaHCO3	0.36
	K_2_HPO_4_ · 3H_2_O	0.23
	HCl (1M)	40 mL
	CaCl_2_	0.29
	Na_2_SO_4_	0.07
	KCl	0.23
	(HOCH_2_)_3_CNH_2_	6.12
	HCl(1M) to stabilize pH	5 mL
Ringer’s solution	NaCl	8.60
	CaCl_2_ · 2 H_2_O	0.48
	KCl	0.30
Artificial saliva	KCl	0.40
	NaCl	0.40
	Na_2_HPO_4_	0.70
	CaCl_2_	0.60
	Na_2_S · 9H_2_O	0.012
	Urea	1.00

**Table 3:** Average contact angle values before and after 56 days of incubation in solutions simulating a physiological environment.

		Water Contact Angle ϴ, (°)			
Sample Name	Before Incubation	After Incubation in PBS	After Incubation in SBF	After Incubation in A. Saliva	After Incubation in Ringer’s
PGS-IPDI	113.7 ± 9.2	120.0 ± 7.7	126.4 ± 8.4	108.1 ± 7.7	125.4 ± 11.4
PGS-IPDI 15HAP_B	111.5 ± 3.4	117.1 ± 5.7	119.8 ± 4.4	102.0 ± 7.7	120.9 ± 5.9
PGS-IPDI 15HAP_ICMB	118.2 ± 8.5	116.2 ± 8.5	125.1 ± 4.0	102.6 ± 9.8	122.9 ± 8.2

**Table 4:** Thermal stability of PGS_IPDI foams before and after 56 days of incubation in degradation solutions.

Sample Name	Mass Loss at 900 °C	T_−5wt%_, (°C)	T_−10wt%_, (°C)	T_−20wt%_, (°C)	T_−50wt%_, (°C)	1st Inflection Point	2nd Inflection Point
PGS-IPDI before incubation	96.9	302.7	320.8	362.8	421.4	325.1	427.7
PGS-IPDI_PBS	95.6	280.0	302.5	344.3	421.6	310.9	426.4
PGS-IPDI_SBF	97.0	281.7	305.9	343.5	420.9	314.5	427.5
PGS-IPDI_A.Saliva	95.8	272.3	296.3	339.7	421.7	305.9	404.9
PGS-IPDI_Ringer’s	97.7	293.4	310.0	338.9	417.2	313.9	426.8
PGS-IPDI/15HAP_B before incubation	81.8	308.8	328.4	379.7	427.9	329.9	426.0
PGS-IPDI/15HAP_B_PBS	80.9	298.9	319.2	367.2	429.4	320.5	428.9
PGS-IPDI/15HAP_B_SBF	83.3	302.0	323.5	374.2	429.2	329.7	428.4
PGS-IPDI/15HAP_B_A.Saliva	82.4	296.2	318.9	364.8	428.7	323.0	428.2
PGS-IPDI/15HAP_B_Ringer’s	85.9	295.3	319.0	376.2	426.7	322.4	425.9
PGS-IPDI/15HAP_ICMB before incubation	84.8	301.9	319.2	370.1	425.2	321.0	426.9
PGS-IPDI/15HAP_ICMB_PBS	82.6	284.2	304.0	365.9	427.5	307.6	426.0
PGS-IPDI/15HAP_ICMB_SBF	83.4	293.2	313.0	375.5	429.3	314.7	428.1
PGS-IPDI/15HAP_ICMB_A.Saliva	81.7	280.1	300.9	374.7	430.4	302.8	405.9
PGS-IPDI/15HAP_ICMB_Ringer’s	83.4	293.2	313.0	375.5	429.3	314.7	428.1

**Table 5:** Glass transition temperatures of PGS-based foams before and after 56 days of incubation in degradation solutions.

Sample Name	T_g_, (°C)	Sample Name	T_g_, (°C)	Sample Name	T_g_, (°C)
PGS-IPDI before	10.07	PGS-IPDI 15HAP_B before	7.72	PGS-IPDI 15HAP_ICMB before	1.58
PGS-IPDI PBS	14.08	PGS-IPDI 15HAP_B PBS	1.24	PGS-IPDI 15HAP_ICMB PBS	3.53
PGS-IPDI SBF	15.20	PGS-IPDI 15HAP_B SBF	1.42	PGS-IPDI 15HAP_ICMB SBF	9.07
PGS-IPDI A. Saliva	23.54	PGS-IPDI 15HAP_B A. Saliva	9.56	PGS-IPDI 15HAP_ICMB A. Saliva	10.87
PGS-IPDI Ringer’s	16.87	PGS-IPDI 15HAP_B Ringer’s	2.42	PGS-IPDI 15HAP_ICMB Ringer’s	2.26

**Table 6:** Theoretical elemental composition of foams depending on the degree of reaction of isocyanate with poly(glycerol sebacate).

Sample Name	C%	H%	O%	N%
PGS	56.52	8.70	34.78	-
IPDI	65.00	8.00	14.00	13.00
PGS:IPDI 1:1	60.24	8.43	25.70	5.62
PGS:IPDI 1:0.75	59.66	8.47	27.12	4.75
PGS:IPDI 1:0.5	58.91	9.82	28.94	3.62

**Table 7:** Experimental elemental compositions of foams after 56 days of incubation.

Sample Name	C%	H%	O%	N%
PGS-IPDI before incubation	60.56	8.55	27.71	3.18
PGS-IPDI after extraction	60.91	8.56	27.21	3.33
PGS-IPDI_PBS	60.79	8.58	27.26	3.37
PGS-IPDI_SBF	62.39	8.79	25.86	2.95
PGS-IPDI_A.Saliva	60.63	8.55	27.41	3.41
PGS-IPDI_Ringer’s	61.09	8.61	26.95	3.35

## Data Availability

The original contributions presented in this study are included in the article. Further inquiries can be directed to the corresponding authors.

## Abbreviations

The following abbreviations are used in this manuscript:

pPGS	Prepolymer of poly(glycerol sebacate)
pPGS-IPDI	Prepolymer of poly(glycerol sebacate) modified by isophorone diisocyanate
HAP_B	Nanometric hydroxyapatite
HAP_ICMB	Micrometric particles of biphasic calcium phosphate
PGS-IPDI	Crosslinked foams prepared from pPGS-IPDI
PGS-IPDI/15HAP_B	Crosslinked foams prepared from pPGS-IPDI and HAP_B
PGS-IPDI/15HAP_ICMB	Crosslinked foams prepared from pPGS-IPDI and HAP_ICMB
SBF	Simulated body fluid
PBS	Physiologically buffered saline
